# Hysteroscopic Removal of Intrauterine Device in Pregnancy: A Scoping Review to Guide Personalized Care

**DOI:** 10.3390/medicina58111688

**Published:** 2022-11-21

**Authors:** Guglielmo Stabile, Francesco Cracco, Luigi Nappi, Felice Sorrentino, Salvatore Giovanni Vitale, Stefano Angioni, Stefania Carlucci, Giuseppe Ricci

**Affiliations:** 1 Department of Obstetrics and Gynecology, Institute for Maternal and Child Health-IRCCS “*Burlo Garofolo*”, 34137 Trieste, Italy; 2 Department of Medicine, Surgery and Health Sciences, University of Trieste, 34137 Trieste, Italy; 3 Departments of Obstetrics and Gynecology and Medical and Surgical Sciences, University of Foggia, 71121 Foggia, Italy; 4 Division of Gynecology and Obstetrics, Department of Surgical Sciences, University of Cagliari, 95121 Cagliari, Italy

**Keywords:** embryoscopy, intrauterine device, intrauterine system, hysteroscopy, pregnancy

## Abstract

*Background and objectives*: Pregnancies that occur with an intrauterine device (IUD) in situ are at increased risk for developing severe conditions which may affect the fetus and the mother. The incidence of such adverse consequences significantly drops after device removal. A scoping review of the literature was performed to highlight the risks, benefits, and outcomes of hysteroscopic removal of intrauterine devices in early pregnancy. *Materials and Methods*: PubMed, Scopus, and Web of Science were searched. The review included all reports from 1990 to October 2022. The research strategy adopted included different combinations of the following terms: (“hysteroscopy”) AND (“pregnancy”) AND (“intrauterine device” or “IUD”) AND (“intrauterine system” or “IUS”). A scoping review of the hysteroscopic removal of IUDs during pregnancy was performed. All studies identified were listed by citation, title, authors, and abstract. Duplicates were identified by an independent manual screening performed by two researchers and then removed. For the eligibility process, two authors independently screened the titles and abstracts of all non-duplicated papers and excluded those not pertinent to the topic. *Results*: PRISMA guidelines were followed. Nine manuscripts were detected, accounting for 153 patients. Most IUD removals occurred during the first trimester of pregnancy. Most of the time, the procedure was safe and without consequences. *Conclusions*: This review highlights the safety and efficacy of operative hysteroscopy as a method of IUD removal in early pregnancy. We recommend using a 3 to 5 mm hysteroscope, avoiding cervical dilation, and maintaining low infusion pressure during the procedure to avoid potential damage to the gestational sac and IUD fragment displacement. Heating the distension media to 30 °C should be considered.

## 1. Introduction

Intrauterine devices (IUDs) represent a commonly used form of contraception, which combines both safety and efficacy. It is established that 14.3% of women of reproductive age choose IUD placement as a form of contraception, especially in Asian countries [1]. Two main different types of IUDs are currently available on the market: copper IUD (Cu-IUD) and levonorgestrel-releasing IUD (also known as an intrauterine system, IUS), which differ in both the mechanism of action and duration [2].

Although IUDs are considered among the most effective contraception methods for pregnancy prevention, they are characterized by a risk of unwanted pregnancy (i.e., failure rate). The failure rate ranges from 0.2% for the levonorgestrel-releasing IUD to 0.8% for the copper IUD (Cu-IUD) [3].

Pregnancies that occur with an IUD in situ are at increased risk for developing severe conditions that may affect the fetus and the mother: ectopic pregnancies, spontaneous abortion in the first or second trimester, preterm delivery, and chorioamnionitis. Such complications might sometimes be severe, requiring demanding and sometimes challenging therapies [4]. In addition, levonorgestrel-releasing IUDs may be responsible for teratogenic risk [5].

With regards to the techniques used to retrieve IUDs during pregnancy in the case of non-visible strings during a gynecological evaluation, ultrasound (US)-guided forceps removal and hysteroscopic methods play an important role, with good success rates reported in the literature [6,7]. Nevertheless, both US removal and hysteroscopy come with a perinatal loss rate that ranges from 35% to 45%, mainly due to the development of complications. However, these rates are significantly lower than the 50% perinatal loss that may occur where pregnancy continues with a retained IUD [6].

Different procedures for hysteroscopic or ultrasound-guided IUD removal have been described in the literature; however, despite significant diagnostic and therapeutic advances made in hysteroscopy [8,9], neither hysteroscopy nor ultrasound-guided forceps removal has been shown to be superior to the other. It is likely that structuring a guideline about how to perform hysteroscopy or forceps removal to retrieve IUDs is limited by the poor literature available and the several differences in terms of technologies and expertise present among the operators [7]. Thus, we performed this review to analyze data regarding the hysteroscopic removal technique to highlight its benefits, risks, and results (pregnancy outcome): a scoping review of hysteroscopic removal of retained IUD during pregnancy was conducted, including the literature available from 1990 to October 2022.

## 2. Materials and Methods

**Figure 1:**
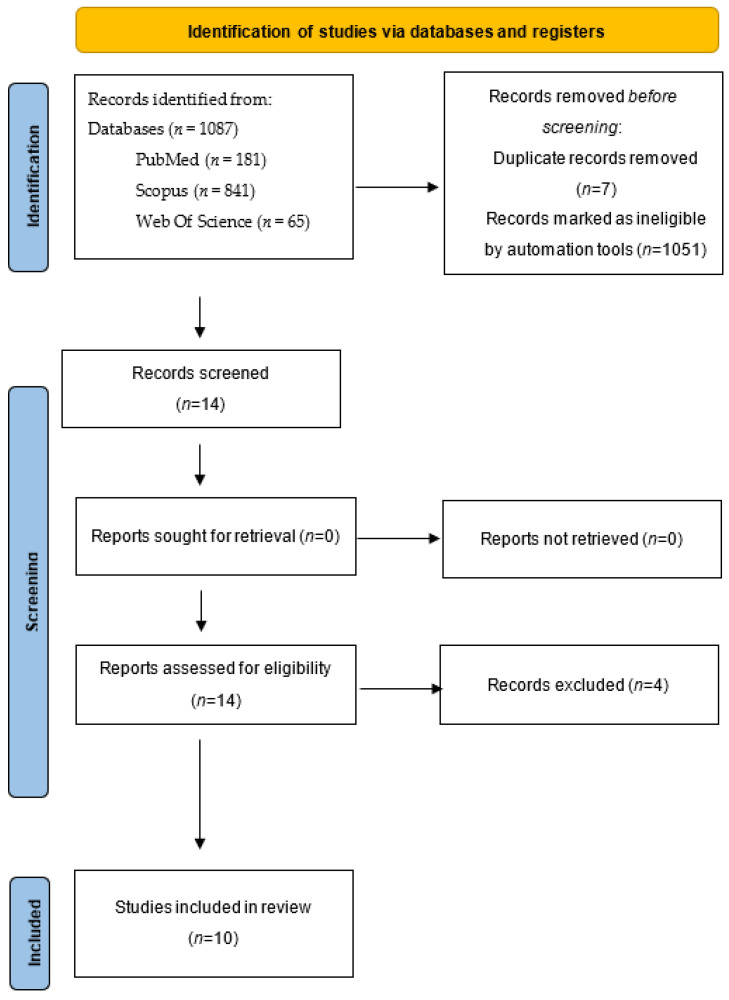
PRISMA flow diagram of the systematic literature search.

We identified relevant original studies in the English language through a search of the MEDLINE, Scopus, and Web of Science (1990 to October 2022) databases using the following terms: (“hysteroscopy”) AND (“pregnancy”) AND (“intrauterine device” or “IUD”) AND (“intrauterine system” or “IUS”). The scoping review was made in accordance with the Preferred Reporting Items for Systematic Reviews and MetaAnalyses (PRISMA) guidelines (http://prisma-statement.org/prismastatement/Checklist, accessed on 2 August 2022). A flow chart of the systematic literature search according to PRISMA guidelines [10] is reported in Figure 1.

Three authors (G.S., F.C., and S.G.V.) independently screened the titles and abstracts of studies obtained by the search strategy. Duplicates were identified through manual screening performed by the same researcher and then removed. The full text of each potentially relevant study was obtained and assessed for inclusion independently by the two authors (G.S. and F.C.). They also independently extracted data from the included studies. Three other authors (F.S., L.N., and G.R.) independently reviewed the selection and data extraction process.

In our review, we examined the patients’ age, gestational age, pregnancy evolution, and, if it was a preterm delivery, neonatal outcome. For each outcome, the mean value and ratio were calculated. We excluded studies with unsuccessful attempts at hysteroscopy in pregnancy. Articles not relevant to the topic were also excluded. All studies identified were examined for the year, citation, title, authors, abstract, and their full texts. 

The results were compared, and any disagreement was discussed and resolved by consensus. Studies with ambiguous or insufficient data, low-quality data, or non-quantifiable outcomes were also excluded.

Two additional studies were found by searching the references lists of the articles previously identified with the research on databases. These two were included in the review. The inclusion of case reports and case series in this review represents a risk of bias. The methodological quality of the included studies was assessed using the Joanna Briggs Institute (JBI) Critical Appraisal Checklist for case reports and case series [11] (see Appendix A—Table A1).

This research was approved by the Institute for Maternal and Child Health IRCCS *Burlo Garofolo* Institutional Review Board (RC 08/2020).

## 3. Results

In total, 126 articles were found through database and reference searches. After the screening process, 13 manuscripts were chosen. Selected cases included those with an intrauterine device, no visible strings, and an evolutive pregnancy in the meantime. Four articles were excluded because they were not relevant to the topic. Therefore, nine studies were included for a total of 153 hysteroscopic removals, as shown in Table 1.

The mean gestational age was 8.2 weeks, even if one study describes a cluster of 8 among 50 patients who had their IUDs extracted after the 12th week of gestation.

Parity was not frequently reported in the included studies. For this reason, we decided not to take parity into consideration as a valuable parameter.

IUD removal was successfully performed in 147 women. After the procedure, the miscarriage rate was 10.2% (15 out of 147 successful procedures). In six women, IUD removal was not successful; all of these pregnancies ended in miscarriage.

Because of the large heterogeneity between all the selected studies, it was not possible to establish an average time interval between the procedure and the miscarriage. However, it is possible to state that cases of abortion after hysteroscopy occur mainly in the first 2 weeks after the procedure (66.7%).

Eighteen patients (12.2%) among all those who underwent an efficacious IUD removal experienced preterm delivery. Unfortunately, many patients were lost to follow-up, and the gestational age at delivery was not known. Additionally, 121 (82.3%) women delivered at the term of pregnancy.

Based on our results, we can state that hysteroscopic removal is possible with a high success rate (96.07%). In all cases in which the procedure failed, there was an abortion (100%). The risk of the procedure is represented by the miscarriage in 10.2% of the cases and preterm delivery in 12.2%. Overall, the outcome was favorable, with an at term pregnancy in 82.3%.

## 4. Discussion

### 4.1. Main Findings

Early pregnancy IUD removal is a challenging procedure, which exposes both the patient and the physician to serious risks, especially when the patient wishes to continue the pregnancy. When IUD strings are not visible at the external os of the cervix, these risks are even higher, so much so that some authors suggest leaving the IUD inside the endometrial cavity to avoid potential miscarriage consequent to the removal procedure [18,19]. In recent years, scientific and technological progress in the field of minimally invasive surgery has allowed the development of new techniques in hysteroscopy [19], which led physicians to attempt endoscopic removal.

Mermet et al. [20] recruited 67 women with Cu-IUDs who wished to continue their pregnancies. The study population was divided into two samples: 38 had the IUD removed, and 29 had the IUD kept in situ. Results showed an increased risk of adverse outcomes in the second group. In the first group, the miscarriage risk rate was 8% (similar to our result), while in the second one it was 48%. Preterm delivery risk and premature rupture of membranes (PROMs) risk was 90% in the retained IUD sample, versus 34% in the women who had their IUDs removed. Furthermore, other complications reported were abnormal vaginal bleeding (AUB), intrauterine infection, and fetal congenital anomalies [20]. This type of complications were described by other authors in the past [21,22].

In a 2011 systematic review, Brahmi et al. [23] examined nine articles and described, a greater risk of adverse pregnancy outcomes (particularly spontaneous abortion, preterm delivery, septic abortion, and chorioamnionitis) in women with retained IUDs than in those who had IUDs removed. The authors concluded that early IUD removal appeared to improve pregnancy outcome, even though it did not eliminate risks.

In 1993, Lin et al. [17] reported 28 cases of successful hysteroscopic IUD removal in 33 women with IUDs in early pregnancy without visible filaments. In two cases, the device was not removed due to unfavorable position, and in three cases no IUD was observed, probably following a spontaneous expulsion of the device. The authors suggested that the examination and removal of IUDs with non-visible filaments in early pregnancy through flexible hysteroscopy is feasible without side effects in women and their fetuses. They also asserted that the removal is easier with earlier gestational age.

### 4.2. Interpretation

Comparing the manuscripts found in our research, it emerges that the miscarriage rate was higher (33% of all abortions) when a hysteroscope of a larger caliber (7 mm) was used than when a hysteroscope with a 3 to 5 mm caliber was used. This may be due to the potential trauma to the uterine tissues and pregnancy caused by the larger caliber and the greater flow of the distension media. These factors may also act on pain and, therefore, on uterine contractility [24], hence modifying the accomplishment of the technique. Furthermore, analyzing the cases found, it is possible to state that cases of miscarriage after hysteroscopy occur mainly in the first 2 weeks after the procedure (66.7%). Later in the pregnancy, the risk is lowered, considering that the risk of miscarriage in the first trimester is generally higher than in the following weeks. When an IUD removal attempt is made in early pregnancy, an outpatient setting such as an office hysteroscopy may be taken into consideration by the physician. In this way, patient anxiety would be reduced, and no anesthetic drugs would be administered.

Regarding IUD displacement in advanced gestational periods (i.e., beyond the 12th gestational week), only a small number of studies are available in the literature, probably since early diagnosis of retained-IUD pregnancy is more common. An analysis of 81 cases by Schiesser et al. [25] described a second-trimester IUD extraction in six pregnancies (7% of the reported cases) as a consequence of prolonged bleeding and severe abdominal pain after an initially conservative approach. In these cases, pregnancy outcomes of second-trimester IUD removal did not differ significantly from those in the first-trimester group, probably due to the small number of cases in the second-trimester group.

A 2018 video article [13] described some tricks to successfully accomplish an IUD removal in early pregnancy, such as preferring small-caliber hysteroscopes and infusing small volumes of isotonic distension media. Four patients underwent IUD removal with success, and all of them delivered live births at term.

Before making any decision, patients must be offered thorough counseling in order to bear the risks of adverse events associated with an ongoing pregnancy with a retained IUD, and to explain the different managements available and their outcomes.

Based on our center’s experience [12], we suggest performing the procedure with caution, preferably avoiding cervical dilation (e.g., using 5 mm hysteroscopes). Low-pressure distension media infusion (50 mmHg during entrance) may help minimize the risk of damage to the gestational sac at the entrance. Subsequently, a further reduction of the pressure (equal to or below 40 mmHg) may reduce the IUD fragment’s mobility, making removal easier. Heating the distension media up to 30 °C, even if not reported in the literature, might represent another tip to reduce vasoconstriction and therefore potential trauma in pregnancy (Table 2).

### 4.3. Strengths and Limitations

The strength of our study is the long period of time overviewed in the literature. All studies selected during the eligibility phase were further evaluated by manual comparison of populations, study settings, and authors to avoid overlapping cases. The main limitation of this review is related to the rarity of this procedure. A second factor may be the review of manuscripts starting in 1990, when some procedures were carried out with rudimentary instruments by operators that might have limited expertise due to the reduced diffusion of the surgical method. Further studies may help enhance patients’ health, both in the contraceptive and reproduction fields.

## 5. Conclusions

IUD hysteroscopic removal in early pregnancies with retained IUD is effective and safe. Patients must be informed of the 10% miscarriage risk and the 12% preterm delivery rate.

The procedure may be preferably performed by a highly experienced hysteroscopist, avoiding cervical dilation, and using a small-caliber hysteroscope (3–5 mm) to minimize potential accidents. Isotonic distension media and reduced flow pressure might help the achievement. The heartbeat of the embryo or fetus should be checked before and after the intervention to assess viability.

## Figures and Tables

**Table 1:** Cases in the literature.

Author (Year) Journal Article Type	Number of Patients (*n*)	Mean Patients Age (Years)	Mean Gestational Age at the Time of the Procedure (Weeks)	Evolutive Pregnancy	Pregnancy Losses (*n*)	Preterm Deliveries	Deliveries at Term	Hysteroscope Caliber (mm)
Stabile G et al. (2022) [12] BMC Women’s Health Case report	1	37	6	1	0 (induced abortion 4 weeks after the procedure)	0	0	5
Ari P. Sanders et al. (2018) [13] Fertility and Sterility Case series	4	34	10.25	4	0	0	4	3–5
Shlomo B. Cohen et al. (2017) [5] JMIG The Journal of Minimally Invasive Gynecology Case series	8	30.5	7.3	7	1 (2 weeks after the procedure)	0	7	5
Ari P. Sanders et al. (2016) [7] Science Direct Case series	25	30.7	11	23	1 (12 days after the procedure)	3	20	5
Perez-Medina et al. (2013) [14] JMIG The Journal of Minimally Invasive Gynecology Case series	7	Not known	8.2	6	1 (2 weeks after retrieval of the device)	0	7	Non available. Operating channel was 1.7 mm in diameter (5F)
McCarthy et al. (2012) [6] Contraception Case report	1	30	8	1	0	0	1	Not available
Rut Aguiar Couto et al. (2008) [15] Prog Obstet Ginecol. Case series	4	31	7	3	1 (7 weeks after the procedure)	0	3	3,9–5,9
Neis K.J. et al. (1994) [16] Gynaecological Endoscopy Case series	26	Not known	8	24	2 (first postoperative day and 6 weeks after the removal)	0	24	Not available
Jen-Ching Lin et al. (1993) [17] Journal of Gynecologic Surgery Case series	28	Not known	7	24	4	0	24	3.7–4.8
Assaf et al. (1992) [18] Contraception Case series	50	Not known	42 under 12 weeks of gestation, 8 patients more than 12 weeks of gestation	46	4 (2 immediate abortions, 2 abortions after a few days)	15	31	7

**Table 2:** Procedural tricks for the hysteroscopic removal of IUD fragments.

Hysteroscopic Procedural Tricks		Rationale
**1**	Use isotonic distension fluids	Minimize gestational sac trauma
**2**	Avoid cervical dilation	Avoid potential gestational sac trauma
**3**	Prefer small-caliber hysteroscopes (e.g., 3 to 5 mm instruments)	Helps to avoid cervical dilation
**4**	Use of low-pressure distension media infusion, during entrance (50 mmHg) and when grasping (40 mmHg)	Minimize gestational sac trauma and reduce IUD fragment mobility
**5**	Consider heating the distension media up to 30 °C	Reduce vasoconstriction

## Data Availability

The authors confirm that the data supporting the findings of this study are available within the article.

## Appendix A

**Table A1:** JBI Critical Appraisal Checklist for Reviews.

Author, Year	Study Type	D1	D2	D3	D4	D5	D6	D7	D8	D9	D10
Stabile G. et al. (2022) [12] BMC Women’s Health	Case report	Yes	Yes	Yes	Yes	Yes	Yes	No	Yes	\	\
Ari P. Sanders et al. (2018) [13] Fertility and Sterility	Case series	Yes	Yes	Yes	Yes	Yes	No	Yes	Yes	No	Yes
Shlomo B. Cohen et al. (2017) [5] JMIG The Journal of Minimally Invasive Gynecology	Case series	Yes	Yes	Yes	Yes	Yes	No	Unclear	Yes	No	Yes
Ari P. Sanders et al. (2016) [7] Science Direct	Case series	Yes	Yes	Yes	Yes	Yes	No	Yes	Yes	No	Yes
Perez-Medina et al. (2013) [14] JMIG The Journal of Minimally Invasive Gynecology	Case series	Yes	Yes	Yes	No	No	No	Unclear	Yes	No	Yes
McCarthy et al. (2012) [6] Contraception	Case report	Yes	Yes	Yes	Yes	Yes	Yes	Yes	Yes	\	\
Rut Aguiar Couto et al. (2008) [15] Prog Obstet Ginecol.	Case series	Yes	Yes	Yes	Yes	Yes	Unclear	Yes	Yes	No	NA
Neis K.J. et al. (1994) [16] Gynecological Endoscopy	Case series	Yes	Yes	Yes	Unclear	Unclear	No	No	Yes	No	Unclear
Jen-Ching Lin et al. (1993) [17] Journal of Gynecologic Surgery	Case series	Yes	Yes	Yes	Yes	Yes	Unclear	No	Yes	No	Unclear
Assaf et al. (1992) [18] Contraception	Case series	Yes	Yes	Yes	Yes	Yes	No	No	Yes	No	NA

**JBI Critical Appraisal Checklist For Case Series**

D1. Were there clear criteria for inclusion in the case series?
D2. Was the condition measured in a standard, reliable way for all participants included in the case series?
D3. Were valid methods used for identification of the condition for all participants included in the case series?
D4. Did the case series have consecutive inclusion of participants?
D5. Did the case series have complete inclusion of participants?
D6. Was there clear reporting of the demographics of the participants in the study?
D7. Was there clear reporting of clinical information of the participants?
D8. Were the outcomes or follow-up results of cases clearly reported?
D9. Was there clear reporting of the presenting site(s)/clinic(s) demographic information?
D10. Was statistical analysis appropriate?

**JBI Critical Appraisal Checklist For Case Reports**

D1. Were patient’s demographic characteristics clearly described?
D2. Was the patient’s history clearly described and presented as a timeline?
D3. Was the current clinical condition of the patient on presentation clearly described?
D4. Were diagnostic tests or assessment methods and the results clearly described?
D5. Was the intervention(s) or treatment procedure(s) clearly described?
D6. Was the post-intervention clinical condition clearly described?
D7. Were adverse events (harms) or unanticipated events identified and described?
D8. Does the case report provide takeaway lessons?

## References

[1] Buhling K.J., Zite N.B., Lotke P., Black K. (2014). Worldwide use of intrauterine contraception: A review. Contraception.

[2] Creinin M., Kohn J.E., Tang J.H., Serna T.B. (2022). Society of Family Planning Committee statement on IUD nomenclature. Contraception.

[3] Luukkainen T., Toivonen J. (1995). Levonorgestrel-releasing IUD as a method of contraception with therapeutic properties. Contraception.

[4] Di Lorenzo G., Romano F., Mirenda G., Cracco F., Buonomo F., Stabile G., Facchin S., Ricci G. (2021). “Nerve-sparing” laparoscopic treatment of parametrial ectopic pregnancy. Fertil. Steril..

[5] Cohen S.B., Bouaziz J., Bar-On A., Schiff E., Goldenberg M., Mashiach R. (2017). In-office Hysteroscopic Extraction of Intrauterine Devices in Pregnant Patients Who Underwent Prior Ultrasound-guided Extraction Failure. J. Minim. Invasive Gynecol..

[6] McCarthy E.A., Jagasia N., Maher P., Robinson M. (2012). Ultrasound-guided hysteroscopy to remove a levonorgestrel intrauterine system in early pregnancy. Contraception.

[7] Sanders A.P., Fluker M.R., Sanders B.H. (2016). Saline Hysteroscopy for Removal of Retained Intrauterine Contraceptive Devices in Early Pregnancy. J. Obstet. Gynaecol. Can..

[8] Vitale S.G. (2020). The Biopsy Snake Grasper Sec. VITALE: A New Tool for Office Hysteroscopy. J. Minim. Invasive Gynecol..

[9] Sorrentino F., De Feo V., Stabile G., Tinelli R., D’Alterio M., Ricci G., Angioni S., Nappi L. (2021). Cesarean Scar Pregnancy Treated by Artery Embolization Combined with Diode Laser: A Novel Approach for a Rare Disease. Medicina.

[10] Page M.J., McKenzie J.E., Bossuyt P.M., Boutron I., Hoffmann T.C., Mulrow C.D., Shamseer L., Tetzlaff J.M., Akl E.A., Brennan S.E. (2021). The PRISMA 2020 statement: An updated guideline for reporting systematic reviews. BMJ.

[11] Munn Z., Barker T.H., Moola S., Tufanaru C., Stern C., McArthur A., Stephenson M., Aromataris E. (2020). Methodological quality of case series studies: An introduction to the JBI critical appraisal tool. JBI Database Syst. Rev. Implement. Rep..

[12] Stabile G., Godina C., Cracco F., Mangino F.P., Canton M., Romano F., Ricci G. (2022). Hysteroscopic removal of intrauterine device in early pregnancy. BMC Womens Health.

[13] Sanders A.P., Sanders B. (2018). Hysteroscopic removal of intrauterine devices in pregnancy. Fertil. Steril..

[14] Pérez-Medina T., Sauco J.S., Ríos M., Pereira A., Argila N., Cabezas E., Cayuela E. (2014). Hysteroscopy in Pregnancy-Related Conditions: Descriptive Analysis in 273 Patients. J. Minim. Invasive Gynecol..

[15] Couto M.R.A., Hernández R.V., Rodríguez M.G., Portela A.L., Valenzuela A.O. (2008). Extracción de DIU mediante histeroscopia en la gestación precoz: Nuestra experiencia en 4 casos. Prog. Obs. Y Ginecol..

[16] Neis K.J., Brandner P., Otte C. (1994). Hysteroscopic removal of lost intra-uterine devices in pregnancy. Gynaecol. Endosc..

[17] Lin J.C., Chen Y.O., Lin B.L., Valle R.F. (1993). Outcome of Removal of Intrauterine Devices with Flexible Hysteroscopy in Early Pregnancy. J. Gynecol. Surg..

[18] Assaf A., Gohar M., Saad S., El-Nashar A., Aziz A.A. (1992). Removal of intrauterine devices with missing tails during early pregnancy. Contraception.

[19] Vitale S.G., Laganà A.S., Caruso S., Garzon S., Vecchio G.M., La Rosa V.L., Casarin J., Ghezzi F. (2021). Comparison of three biopsy forceps for hysteroscopic endometrial biopsy in postmenopausal patients (HYGREB-1): A multicenter, single-blind randomized clinical trial. Int. J. Gynecol. Obstet..

[20] Mermet J., Bolcato C., Rudigoz R.C., Dargent D. (1986). Outcome of pregnancies with an intrauterine devices and their management. Rev. Fr. Gynecol. Obstet..

[21] Tatum H.J., Schmidt F.H., Jain A.K. (1976). Management and outcome of pregnancies associated with the Copper T intrauterine contraceptive device. Am. J. Obstet. Gynecol..

[22] Alvior G.T. (1973). Pregnancy outcome with removal of intrauterine device. Obstet. Gynecol..

[23] Brahmi D., Steenland M.W., Renner R.-M., Gaffield M.E., Curtis K.M. (2012). Pregnancy outcomes with an IUD in situ: A systematic review. Contraception.

[24] Sorrentino F., Petito A., Angioni S., D’Antonio F., Severo M., Solazzo M.C., Tinelli R., Nappi L. (2021). Impact of anxiety levels on the perception of pain in patients undergoing office hysteroscopy. Arch. Gynecol. Obstet..

[25] Schiesser M., Lapaire O., Tercanli S., Holzgreve W. (2004). Lost intrauterine devices during pregnancy: Maternal and fetal outcome after ultrasound-guided extraction. An analysis of 82 cases. Ultrasound Obstet. Gynecol..

